# Supplementary material for: Anthropogenic remediation of heavy metals selects against natural microbial remediation
Source: Proc Biol Sci. 2019 Jun 19;286(1905):20190804. doi: 10.1098/rspb.2019.0804 (PMC6599979; doi:10.1098/rspb.2019.0804)

Supplementary Information.

Table S1. Mean standardized concentration of individual metals in control and limed soil microcosms. The final column summarizes the results of one-tailed t-tests comparing standardized treatment differences to zero, corrected for multiple testing (in italics). Metals are listed from rare to most common.

Heavy metal	Standardized mean [95% CI]		P-value (a | *b**)
	Control	Lime	
Ag	0.499 [0.006, 0.993]	1.484 [0.260, 2.708]	0.932 | *0.932*
Cd	0.948 [-0.145, 2.042]	1.053 [0.583, 1.523]	0.553 | *0.671*
Cr	1.170 [0.244, 2.096]	0.830 [0.484, 1.176]	0.249 | *0.456*
Co	1.103 [0.182, 2.023]	0.897 [0.539, 1.523]	0.338 | *0.532*
Mn	1.333 [0.518, 2.148]	0.678 [0.347, 1.009]	0.029 | *0.068*
**Cu**	**1.150 [0.880, 1.421]**	**0.845 [0.768, 0.951]**	**0.009 | *0.047***
Zn	1.161 [0.705, 1.617]	0.839 [0.639, 1.062]	0.031 | *0.068*
Ni	0.964 [0.569, 1.359]	1.036 [0.694, 1.378]	0.610 | *0.671*
**Al**	**1.233 [0.714, 1.752]**	**0.767 [0.375, 1.159]**	**0.006 | *0.047***
Mg	1.123 [0.670, 1.588]	0.871 [0.552, 1.190]	0.014 | *0.052*
Fe	0.976 [0.403, 1.548]	1.024 [0.643, 1.406]	0.560 | *0.671*

* P-values were corrected for multiple testing (*'p.adjust'* function with method = *fdr*).

Figure S1. Relative abundance of common phyla before (T_0) and after experimental manipulation: microbial communities were incubated for 12 weeks in paired soil microcosms that were either supplemented with (L) a single dose of hydrated lime (L) or an equal volume of sterilized *dd*H20 (C).

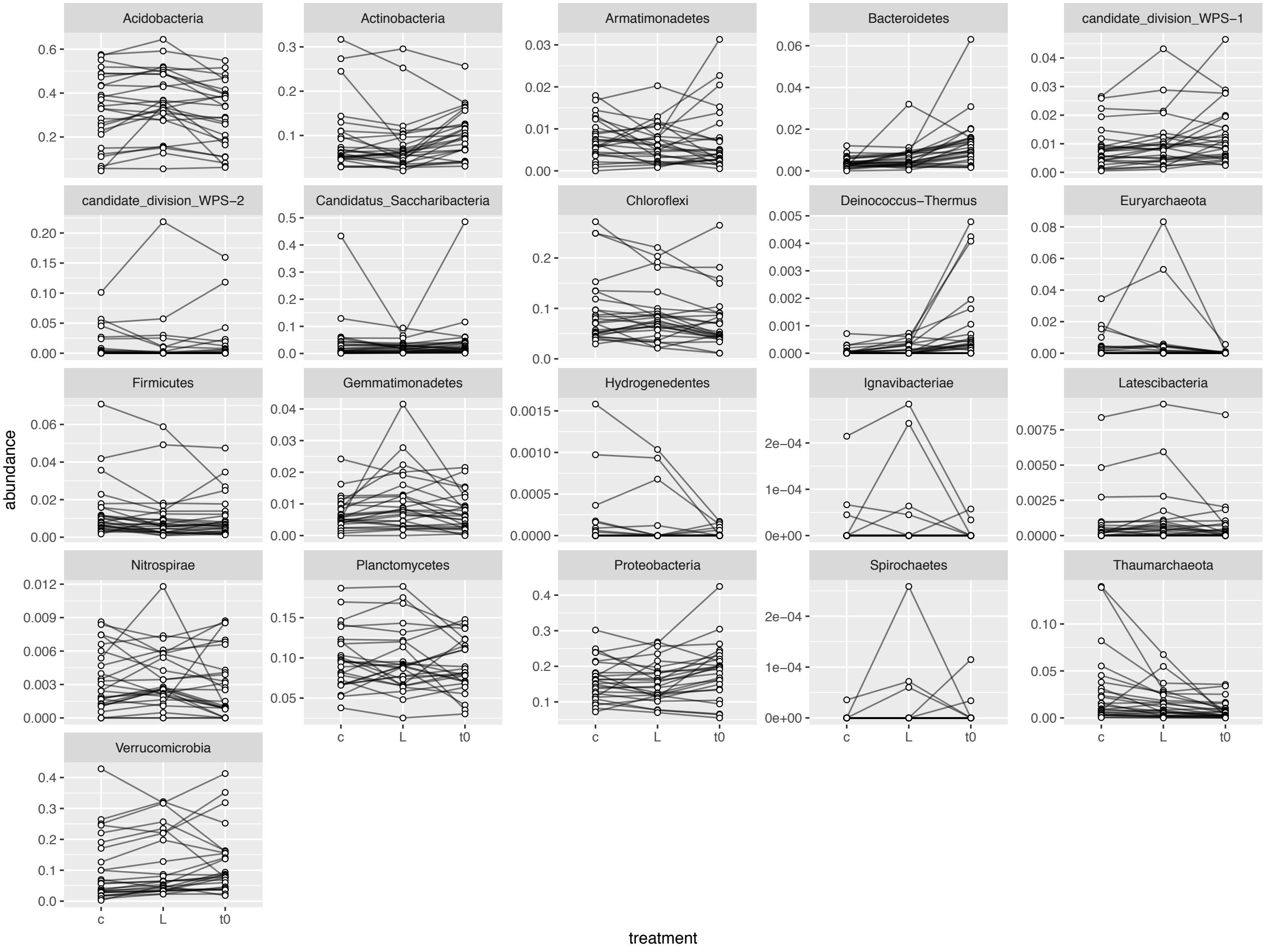

Supplement: Supplementary Information [file rspb20190804supp1.pdf]
